# Managing Coagulation Abnormalities, Bleeding, and Thrombosis in Patients with Cirrhosis

**DOI:** 10.1007/s11606-026-10478-4

**Published:** 2026-05-07

**Authors:** Armando Tripodi, Vincenzo La Mura, Fabio Piscaglia, Bernardo Stefanini, Nicoletta Riva, Walter Ageno

**Affiliations:** 1 IRCCS Ca’ Granda Maggiore Hospital Foundation, Angelo Bianchi Bonomi Hemophilia and Thrombosis Center, Milan, Italy; 2 Division of Internal Medicine, Hepatobiliary and Immunoallergic Disease, IRCCS Azienda Ospedaliero-Universitaria di Bologna, Bologna, Italy; 3 Department of Medical and Surgical Sciences, University of Bologna, Bologna, Italy; 4 Department of Pathology, Faculty of Medicine and Surgery, University of Malta, Msida, Malta; 5 Department of Medicine, University of Padua, Padua, Italy

**Keywords:** cirrhosis, laboratory, bleeding, portal vein thrombosis, atrial fibrillation, anticoagulation

## Abstract

Cirrhosis is associated with a narrow balance between procoagulant and anticoagulant factors that may lead to potentially serious complications. Interpretation of laboratory tests, prevention of bleeding during invasive procedures, and use of anticoagulant drugs for the prevention and treatment of thromboembolism are often challenging. After reviewing the most contemporary literature, we hereby provide guidance to navigate the evidence and support clinical decisions. Based on current knowledge, prothrombin time and activated partial thromboplastin time do not accurately describe hemostasis in patients with cirrhosis and should not be used to predict bleeding. Rather, a careful assessment of patient and procedure-related variables better helps to identify patients at increased bleeding risk. Because procedure-related bleedings are uncommon in patients with cirrhosis, the use of prophylactic strategies is seldom necessary in daily practice. In case of perioperative bleeding, viscoelastometry may be useful to drive decisions on the use of transfusion products. Portal vein thrombosis is a common complication in patients with cirrhosis and requires a timely start of anticoagulant treatment, especially when vessel obstruction exceeds 50% of the lumen diameter. Treatment should be continued for at least 6 months. The direct oral anticoagulants are increasingly used in this setting, representing a valid alternative to the heparins and vitamin K antagonists. Atrial fibrillation in cirrhosis is associated with a high risk of ischemic stroke and treatment-related major bleeding. The benefit of anticoagulants is supported by the results of observational studies, and the direct oral anticoagulants are suggested as the first line of treatment also for this population. Clinical trial number: not applicable

## INTRODUCTION

Patients with cirrhosis are at increased risk of bleeding and thrombotic complications due to impaired balance of coagulation factors, altered hemodynamics of the portal vein system, and concomitant conditions, including primary liver cancer. Practicing clinicians face several challenges in the care of these patients, including the interpretation and management of altered laboratory tests, the prevention of bleeding when invasive procedures are needed, and the administration of antithrombotic drugs for the treatment of portal vein thrombosis (PVT) or for stroke prevention in patients with concomitant atrial fibrillation (AF). These situations are expected to increase in the near future, given the rising prevalence of metabolic dysfunction–associated steatotic liver disease–associated cirrhosis, which is also associated with cardiovascular diseases. ^1^ In this article, we provide a non-systematic overview of the most recent evidence from the literature and practical guidance based on expert opinion on the management of these challenging conditions.

## PATHOPHYSIOLOGY OF HEMOSTASIS IN PATIENTS WITH CIRRHOSIS AND INTERPRETATION OF LABORATORY TESTS

Coagulation is a complex and tightly regulated system, whereby the procoagulant factors that favor thrombin production and fibrin formation are balanced by the anticoagulant factors. Cirrhosis was long considered as the epitome of the acquired hemorrhagic diseases and the bleeding events that were occasionally observed were attributed to the abnormalities of the basic laboratory tests, namely prothrombin time (PT) and activated partial thromboplastin time (aPTT). Thrombocytopenia and thrombocytopathy that also characterize cirrhosis were considered as contributors to bleeding. Moreover, patients with more severe coagulation abnormalities had also the highest risk of variceal bleeding. ^2^

**Figure 1:**
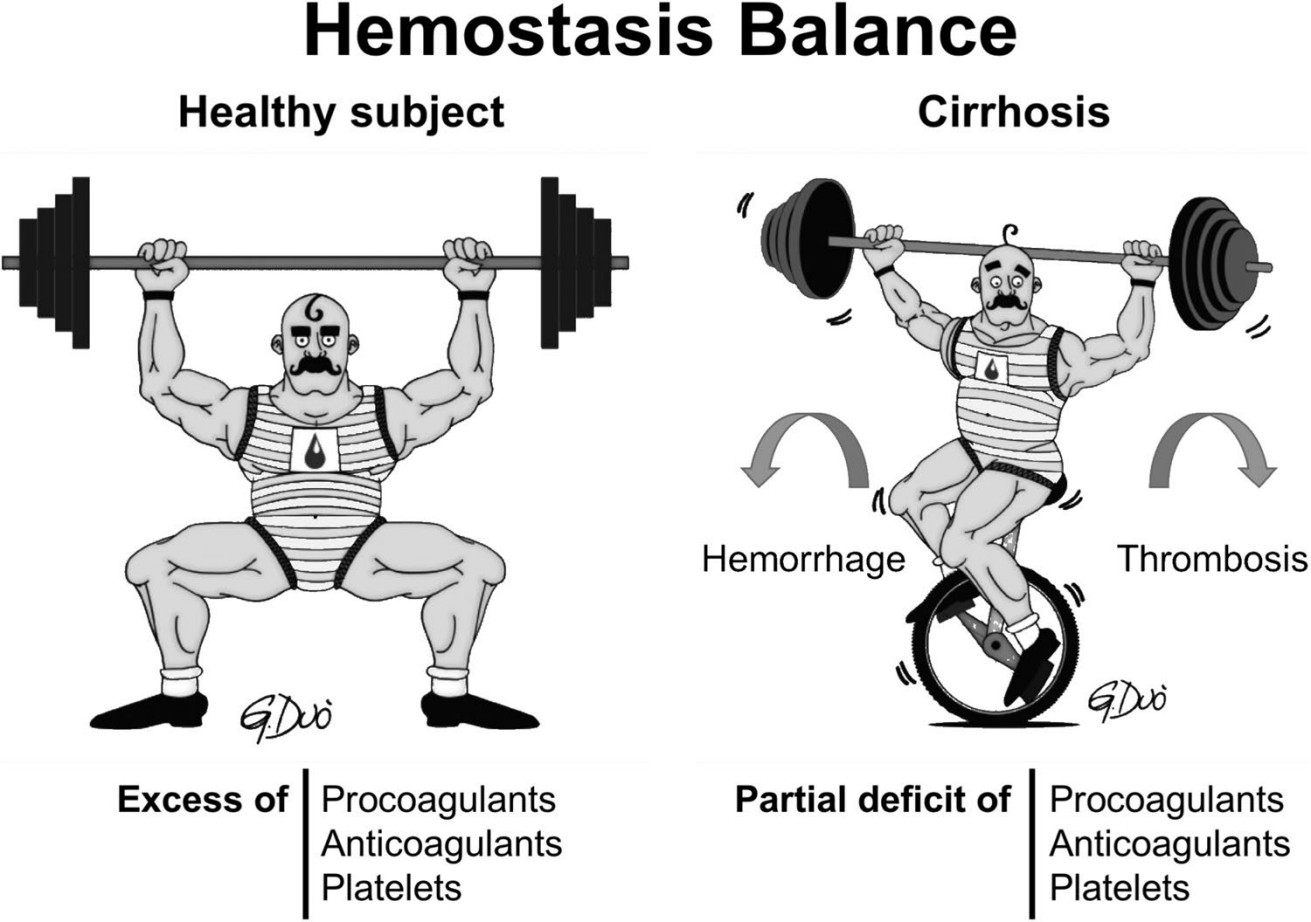
Schematic representation of the hemostatic balance. Healthy subjects possess a stable hemostatic balance, as they possess activities of pro- and anticoagulant factors that are in excess of their needs. Indeed, for a bleeding or thrombotic event to occur, the activity of pro- or anticoagulant factors must be reduced to levels below 50%. In cirrhosis, there is partial deficiency of procoagulants, anticoagulants, and platelets. Hence, patients have a restored hemostatic balance, which is, however, unstable and may occasionally tip toward thrombosis or hemorrhage, depending on the circumstantial risk factors. Permission was obtained from the publisher of *Blood Transfusion*, where the figure was published with the article: Tripodi A., Chantarangkul V., Primignani M. Management of hemostasis abnormalities in cirrhosis: from bench to bedside. *Blood Transfus* 2016; 14 (Suppl 5): LE11. https://doi.org/10.2450/2016.S5.

More recently, the understanding of the pathophysiology of hemostasis in cirrhosis underwent dramatic changes. ^3^ In vitro experiments provided evidence that both coagulation and primary hemostasis are rebalanced, though unstable, and may paradoxically tip toward hemorrhage or thrombosis depending on circumstantial risk factors (Fig. 1). ^3^

In 2005, it was postulated that the abnormalities of the PT/aPTT observed in patients with cirrhosis reflect the reduced synthesis of procoagulant factors, but not the parallel reduction of the naturally occurring anticoagulants (antithrombin and protein C). ^4,5^ Hence, PT and aPTT do not reflect the balance of coagulation between pro- and anticoagulants operating in vivo. It was also demonstrated that the generation of thrombin (i.e., the key enzyme of coagulation) is normal if assessed by methods that account for both pro- and anticoagulants. ^4–7^ The apparent paradox of prolonged PT/aPTT and normal thrombin generation is explained by the fact that protein C, to become a coagulation inhibitor, must be activated. The activation is mediated by thrombomodulin, a receptor located on endothelial cells. Plasma and reagents needed to perform PT/aPTT do not contain sufficient amounts of thrombomodulin and therefore PT/aPTT cannot account for the reduced levels of protein C, typically observed in cirrhosis. In contrast, the addition of exogenous thrombomodulin to the test system makes the modified thrombin generation procedure suitable to account for the balance of coagulation, which is operating in vivo. ^8^

A year later, it was also shown that the defective primary hemostasis (i.e., platelet-vessel wall interaction) is rebalanced in cirrhosis, mainly due to the increased levels of the adhesive protein von Willebrand factor that characterizes these patients. ^9^ Von Willebrand factor plays a key role in primary hemostasis facilitating the adhesion and aggregation of platelets.

This mismatch between in vivo and in vitro hemostasis is probably the reason why the basic tests of hemostasis are not good predictors of bleeding in patients with cirrhosis. This hypothesis is supported by clinical observations: no association between PT prolongation on peripheral blood and bleeding from liver during laparoscopic biopsy, ^10^ no association between abnormalities of coagulation and transfusion requirements in patients undergoing liver transplantation, ^11^ and lack of bleeding control in patients with variceal rupture or undergoing hepatectomy by potent procoagulant agents (namely, recombinant activated FVII). ^12,13^

Other tests that were used to predict bleeding in patients with cirrhosis are summarized in Table 1. Whole-blood viscoelastometry may be useful to make decisions on the transfusion product(s) in case of perioperative bleeding, much less to predict bleeding in patients undergoing invasive procedures. Thrombin generation performed on plasma does not accurately predict bleeding, while thrombin generation performed on whole blood seems to be more promising in this respect when performed in severely decompensated patients. ^14^ However, experience with this test is limited and there are no commercially available kits to be used in general clinical laboratories.

**Table 1:** Characteristics and Potential Usefulness of Hemostasis Tests, Currently Used in Cirrhosis

Name	Characteristics	Usefulness
PT	• Tissue factor induced coagulation time	• No prediction of bleeding in patients undergoing surgery or invasive procedure • Useful as prognostic index
aPTT	• Phosphatidyl-serine/contact activators coagulation time	• No prediction of bleeding in patients undergoing surgery or invasive procedure
Light aggregometry	• Aggregometry on platelet-rich plasma activated by various agonists	• Although abnormal in most patients, it does not represent the in vivo function of platelets because it is a static test for which there is no contribution of the VWF
Platelet count	• Counting on autoanalyzers	• There is no consensus on the platelet threshold to be used for transfusion • There is no laboratory method to help making decision on threshold • Current guidelines recommend to consider only severe thrombocytopenia when all the other causes (including pseudo-thrombocytopenia) have been evaluated
Skin bleeding time	• Standardized skin incision on the forearm • Sensitive to thrombocytopenia (platelet count < 100 × 10^9^/L) and thrombocytopathy	• It is the only test operated in vivo • Difficult to be performed. Results are variable and operator dependent
Platelet factor assay (PFA-100)	• In vitro test, mimicking the skin bleeding time • Blood is forced to pass through a membrane coated with platelet agonists • The endpoint is the closure time when blood stop flowing	• Sensitive to VWF • There is no definitive evidence that PFA-100 is sensitive to thrombocytopathy
TM-modified thrombin generation	• In vitro activation of coagulation by means of calcium chloride, small amounts of tissue factor in the presence of exogenous TM and negatively charged phospholipids	• It is the only test that accounts for both pro- and anticoagulants. Thus, represents the balance of pro- and anticoagulants operating in vivo • The only retrospective study available, concludes that it is not a predictor of bleeding in patients with cirrhosis • The variant of the above method performed on whole blood seems promising as a predictor of bleeding in patients with severe decompensation • There are no commercial kits available for the method on whole blood and the experience is scanty
Viscoelastometry	• Currently there are two devices available: RoTem and TEG • Whole blood is activated by calcium chloride, tissue factor and phospholipids, • The viscoelastic properties of clotting blood are recorded by a typical tracing showing various parameters associated with clot formation (velocity and strength)	• Although widely used as a predictor of bleeding in patients undergoing liver transplantation or invasive procedures • It did prove effective in liver transplantation, but not to predict bleeding during invasive procedures • It may be useful to make decision on the type of transfusional product to be used during active bleeding, but there is no evidence from ad hoc trials to assess its prediction of bleeding
Anti-FXa	• In vitro test to monitor unfractionated heparin or low molecular weight heparin (LMWH)	There is no evidence that the assay can accurately be used to measure therapeutic efficacy of heparin infusions in cirrhosis. In vitro studies showed that the anti-FXa assay underestimates LMWH in cirrhosis because of a laboratory artifact (https://doi.org/10.1111/j.1478-3231.2011.2011.2011.02489.x.). The current practice is to use LMWH at fixed dose, adjusted for body weight. Whether other anticoagulants may be useful is unknown

*PT* prothrombin time, *aPTT* activated partial thromboplastin time, *VWF* von Willebrand factor, *TM* thrombomodulin

In vitro platelet aggregometry is often abnormal in cirrhosis, but without the contribution of von Willebrand factor it is unable to capture the platelet function operating in vivo. Promising in this respect might be the new microfluidic technology, which operates at relatively high shear stress. ^15^ As a future perspective, steps should be taken to develop and commercialize more useful laboratory tests for this population.

While currently available laboratory tools are not recommended by international guidelines, ^16^ the blood count when the platelet number is extremely low could be a possible exception. However, the threshold for intervention (i.e., platelet transfusion) is not precisely known, as there are neither clinical trials nor suitable laboratory tests to rely upon. Hence, intervention should be decided on a case-by-case basis. In a study conducted in patients with platelet count < 50 × 10^9^/L undergoing variceal ligation, transfusion of one single adult platelet unit resulted in a small increase in platelet count without normalizing thrombin generation in ex vivo platelet-rich plasma or whole-blood thromboelastometry. ^17^ Furthermore, physicians should be aware of patients for whom thrombocytopenia is not due to cirrhosis and of the many instances—recently described ^18^—of pseudo-thrombocytopenia when platelets are counted on regular autoanalyzers.

## THE MANAGEMENT OF HEMOSTASIS BEFORE INVASIVE PROCEDURE

Changes of traditional hemostatic tests along with the progression of chronic liver disease have fostered for decades the idea that patients with cirrhosis need to have primary and secondary hemostasis and/or fibrinolysis corrected before an invasive procedure. Although this approach has been recently questioned, ^19^ large amounts of blood products are still used as pre-procedural prophylaxis. ^20–23^

Procedure-related bleeding in cirrhosis may range from 2 to 20%. ^24–29^ This wide interval may be explained by the design of the studies (mostly retrospective), the inclusion of non-surgical and surgical procedures, the non-uniform outcome definitions, and the heterogeneity of patient populations. To overcome this uncertainty, a large prospective, international study was recently conducted in 1187 patients with decompensated cirrhosis who underwent 3006 non-surgical procedures during hospitalization. ^28^ The study identified 93 procedure-related bleeding complications (3.1%) in 82 patients (6.9%). Bleedings occurred after dental extractions (36.3%), common bedside procedures (2.9%), vascular interventions (4.4%), endoscopic interventions (1.7%), and percutaneous interventions (5.7%). Patients with a bleeding history had more advanced chronic liver disease or organ dysfunction and were more commonly exposed to high-risk or multiple invasive procedures. MELD score and body mass index were independently associated with bleeding, with body mass index > 40 kg/m^2^ and MELD > 25 being the cut-offs at the highest risk of at least one procedure-related bleeding. Neither platelet count nor PT predicted adverse outcomes. These findings suggest that procedure-related bleeding complications are uncommon for patients with cirrhosis, supporting a reactive rather than a prophylactic management in daily practice.

The other two key issues are represented by the classification of the procedures as high versus low risk of bleeding and the value given by clinicians to the most traditional laboratory tests. It is generally accepted that high-risk procedures are those with an estimated bleeding probability over 1.5% or when bleeding can lead to permanent organ damage or death. ^16^ Nevertheless, discrepancies in risk categorization among guidelines are observed. For instance, while EASL classifies the bleeding risk of both transjugular and percutaneous liver biopsy as low, the American Association for the Study of Liver Disease classifies both as high, and the International Society on Thrombosis and Haemostasis classifies the former as low and the latter as high. ^30–32^ In a recent survey involving 72 experts from Europe, the USA, and Asia, a consensus (≥ 75% agreement) was reached for 52 of 80 procedures (65%). ^29^ Those without agreement included the placement of a transjugular intrahepatic portosystemic shunt, esophagogastroduodenoscopy with band ligation, liver biopsy, and dental extractions. Regarding the laboratory thresholds considered acceptable by the experts before an invasive procedure, agreement was reached on the lowest acceptable platelet count (30 × 10^9^/L and 50 × 10^9^/L for low- and high-risk procedures, respectively), on the discontinuation of aPTT measurement for any procedure, on the discontinuation of the PT-INR measurement for low-risk procedures, and on the lowest acceptable levels of fibrinogen (i.e., 60 mg/dL for low-risk procedures, 100 mg/dL for high-risk procedures, and 120 mg/dL for surgery).

**Figure 2:**
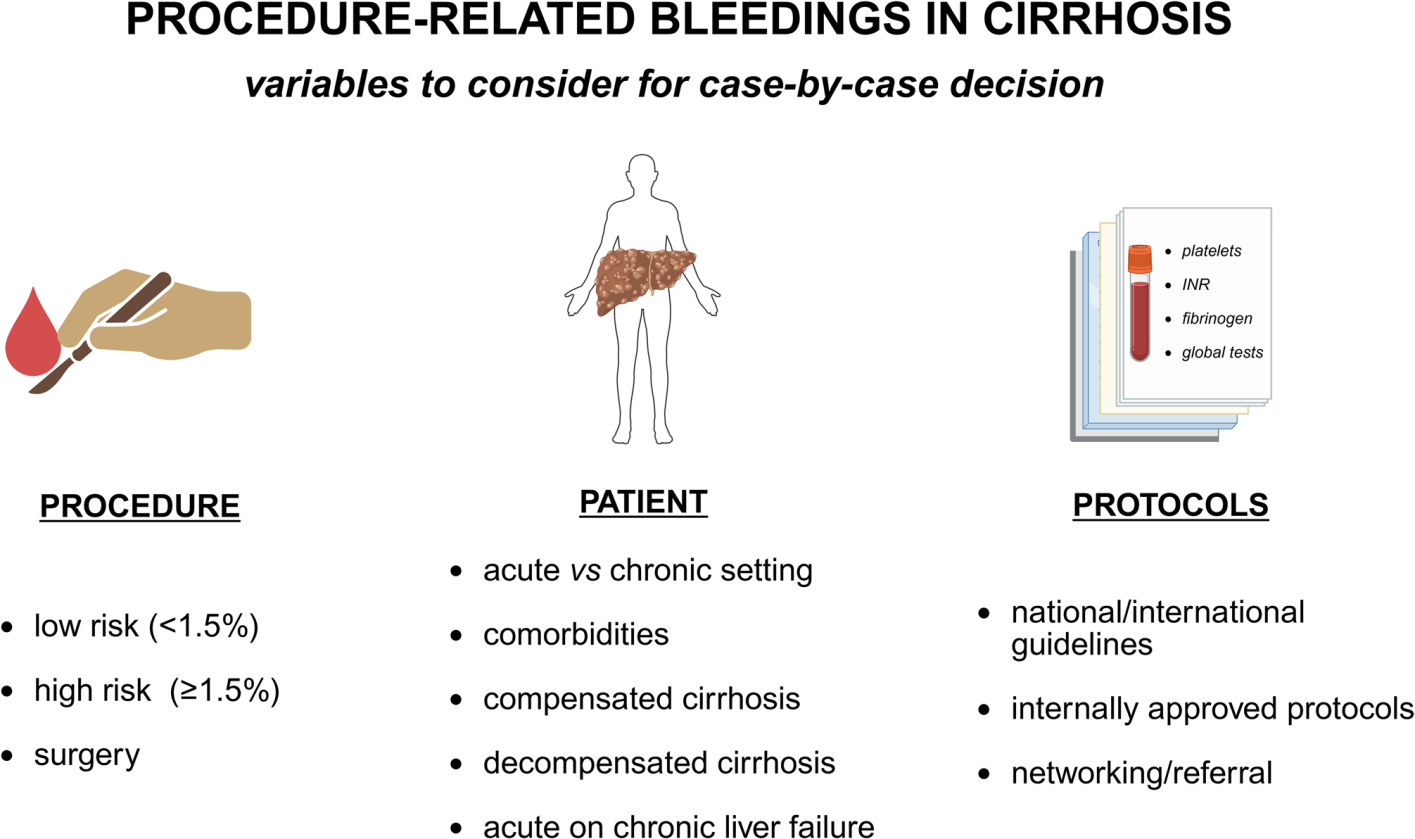
Variables that can influence the case-by-case decision for the management of hemostasis in patients with cirrhosis before an invasive procedure (created with BioRender)

Waiting for large studies to support evidence-based protocols of care, clinicians need to take case-by-case decisions weighing procedure-related risks and patient characteristics following local protocols usually based on expert recommendations (Fig. 2). A widespread use of global laboratory tests that overcome the limitations of currently available assays is also warranted. ^33–35^

**Table:** 

KEYPOINTS for the management of hemostasis before invasive procedures • International guidelines do not recommend the routine use of laboratory monitoring and management of hemostatic factors before invasive procedures, but suggest to consider decisions on a case-by-case basis in special settings • The bleeding risk associated with invasive procedures, the severity of the liver disease, along with the burden of comorbidities should guide this individualized decision • New randomized controlled trials for laboratory-based clinical-decision algorithms should be conducted to implement the current consensus international guidelines • The development of local protocols should be pursued to warrant adequate management of hemostasis and possible referral of selected patients with cirrhosis requiring high-risk invasive procedures	

## PORTAL VEIN THROMBOSIS IN CIRRHOSIS: CLINICAL IMPLICATIONS AND MANAGEMENT

PVT is a frequent and challenging complication in patients with cirrhosis. Its prevalence and incidence are reported heterogeneously due to variability in study populations, including differences between compensated and decompensated cirrhosis and the inclusion of patients with comorbidities known to increase thrombotic risk. A meta-analysis of 74 studies estimated a pooled prevalence of 13.9% (95% CI, 11.2–16.9%), ^36^ while prospective studies report an annual incidence between 2 and 10%. ^37^ The risk of PVT rises with worsening hepatic function, highlighting the interplay between altered portal hemodynamics, endothelial injury, and the unstable, variable coagulation state of cirrhosis. ^37^

As cirrhosis was long regarded as a bleeding disorder, physicians had often a greater fear of hemorrhagic complications than a concern for unresolving PVT. ^38^ Consequently, PVT management has been approached more cautiously and conservatively than other non-life-threatening common complications such as ascites or encephalopathy. However, growing evidence indicates that anticoagulant therapy not only promotes recanalization but also improves survival and transplant outcomes, thus progressively shifting the clinical paradigm. ^39^ In practice, physicians are often confronted with three fundamental questions when managing patients with cirrhosis and PVT: what is the expected benefit of treatment, what are the consequences of withholding it in any given individual patient, and what risk would the patient face if anticoagulated. Response to these questions leads to the decision whether anticoagulation should be started and, ultimately, which therapeutic strategy should be preferred in terms of agents and treatment duration.

There is a solid rationale to support the treatment of PVT, given its association with increased mortality, progression of portal hypertension, technical difficulties during liver transplantation, and reduced post-transplant survival. Achieving resolution and recanalization—whenever feasible and sufficiently safe—is therefore a key therapeutic objective. Although up to 30–40% of patients may experience spontaneous partial or complete recanalization within 6 months, ^40–42^ single-center studies and meta-analyses demonstrated better outcomes with anticoagulation in terms of recanalization. ^40^ Notably, most studies reported data about selected patient populations and focused mainly on the course of PVT. However, patients with cirrhosis constitute a quite fragile population, often with low platelet count and with unstable coagulative balance, making thrombus improvement a likely insufficient proxy of global benefit. Harder endpoints and particularly overall survival appear warranted. Accordingly, the IMPORTAL study, a large individual patient data meta-analysis, tried to answer this question by collecting information on 500 patients with cirrhosis. It showed that anticoagulation significantly improved recanalization rates compared with no anticoagulation. ^43^ Moreover, benefits extended beyond thrombus resolution: anticoagulation reduced both all-cause and liver-related mortality, regardless of baseline liver function or recanalization status, thus providing robust evidence for its benefits. ^43^ However, studies included in the meta-analysis were mainly retrospective, potentially introducing a bias related to confounding factors—such as healthier patients being more likely to receive anticoagulation—that might have impacted the results. However, this risk was partially mitigated by the use of individual patient data which allows for adjustment for covariates considered to have prognostic significance.

Although anticoagulation seems to offer clear benefits, it is not routinely indicated. International guidelines recommend treatment particularly in patients with recent (< 6 months) PVT, with an involvement of at least 50% of the vessel lumen, or in those who are symptomatic or candidates for liver transplantation. ^37,44,45^ These thresholds are justified by clinical evidence suggesting a favorable spontaneous course for PVT with limited extension ^46^ and by hemodynamic principles, as Poiseuille’s law indicates that a 50% luminal narrowing reduces blood flow by approximately 94%. Yet, the management of partial, non-occlusive thrombosis remains controversial. While some clinicians adopt a watchful waiting strategy with close follow-up, emerging data suggest that early anticoagulation may improve outcomes without significantly increasing bleeding risk, thus challenging traditional thresholds and potentially expanding the population of patients with cirrhosis who may benefit from treatment.

Patient selection strategies therefore should aim to balance the probability of recanalization against the risk of bleeding. The prevention of bleeding is related to the presence of esophageal varices at risk, based on the use of non-selective beta-blockers and variceal band ligation. In case of (recurrent) bleeding or recurrent PVT, transjugular intrahepatic portosystemic shunt (TIPS) placement should be considered. While high-quality prospective data are lacking, retrospective studies consistently show no significant increase in major bleeding with careful patient selection. ^40,41,47^

The choice of the optimal anticoagulant remains debated. Low molecular weight heparin (LMWH) is the most extensively studied and guideline-endorsed option, but its use may become problematic in case of extended treatment courses (i.e., beyond 6 months) in terms of therapeutic adherence, probably due to the parenteral route of administration. A prospective observational study reported a discontinuation rate of 21% after a median time of 3 months in patients with cancer treated with LMWH and followed up until 6 months. ^48^ Fondaparinux is also occasionally used, but its long half-life suggests the need for great caution, especially in patients with impaired renal function. Vitamin K antagonists (VKAs) have been largely used and have been shown to be effective and safe in well-selected patients, but PT-INR monitoring can be problematic or even misleading in patients with advanced hepatic dysfunction. Direct oral anticoagulants (DOACs) are increasingly used in clinical practice, with growing evidence supporting their safety in patients with Child-Pugh A cirrhosis and cautious use in Child-Pugh B, while they remain contraindicated in Child-Pugh C. ^49,50^ In the absence of head-to-head comparisons among available drugs, treatment decisions remain largely empirical and individualized.

Once treatment is started, current guidelines recommend reassessment after 3 months of anticoagulant therapy. If partial or complete regression is documented, continuation up to at least 6 months is advised ^37,44^, since thrombosis recurrence was reported in > 40% of cases after discontinuation of anticoagulation. ^41^ In transplant candidates, anticoagulation should generally be maintained until transplantation, whereas in non-transplant patients, discontinuation may be considered after 6–12 months if complete recanalization is achieved, especially if bleeding risk exceeds the benefit of treatment.

Tumor portal vein invasion in hepatocellular carcinoma (HCC) does not represent an indication to anticoagulation, which remains unjustified. Conversely, in patients with PVT and concurrent presence of HCC, anticoagulation should be considered as effective as in patients with cirrhosis without HCC, although global prognosis, concomitant treatment strategies, and the need for interventional procedures should be considered at the time of the therapeutic decision. ^51^

**Table:** 

KEYPOINTS for the management of PVT in patients with cirrhosis • Anticoagulant treatment significantly improves recanalization and reduces mortality in patients with cirrhosis • Low molecular weight heparins are the most widely used anticoagulants for the initial treatment. Increasing evidence supports the safety of direct oral anticoagulants in eligible patients • Anticoagulant treatment should be continued for at least 6 months	

## ANTICOAGULANT THERAPY IN PATIENTS WITH CIRRHOSIS AND ATRIAL FIBRILLATION

AF, the most common cardiac arrhythmia, has a prevalence of 6.6–14.2% in patients with cirrhosis ^52,53^—which is higher than the prevalence reported in the general population—and increases with the severity of liver disease. ^54^

Cirrhosis and AF share several risk factors, such as alcohol consumption and metabolic syndrome components (e.g., hypertension, obesity, diabetes mellitus), ^55,56^ which predispose both to AF and to metabolic dysfunction–associated steatotic liver disease, an emerging cause of cirrhosis. ^57^

The presence of AF in patients with cirrhosis is linked to higher mortality rates, compared to patients with cirrhosis without AF ^51,58^ and to an increased risk of ischemic stroke, hemorrhagic stroke, venous thromboembolism, gastrointestinal hemorrhage, and subdural hemorrhage. ^59^

In order to estimate the thromboembolic risk of non-valvular AF (NVAF), the latest European Society of Cardiology guidelines recommend using the CHA_2_DS_2_-VA score. ^60^ Oral anticoagulation is suggested in patients with CHA_2_DS_2_-VA score 1 and is recommended in patients with CHA_2_DS_2_-VA score ≥ 2. ^60^ Although the HAS-BLED score was previously used to estimate the bleeding risk, the latest ESC guidelines do not recommend using bleeding risk scores to decide on the use of anticoagulant drugs. Instead, guidelines suggest managing individual, modifiable bleeding risk factors. ^60^ Indeed, cirrhosis itself is an independent bleeding risk factor and the bleeding risk (expressed by the HAS-BLED score) was found to correlate with the severity of liver disease (expressed by the Child-Pugh score). ^61^

DOACs are the first line of anticoagulant treatment in patients with NVAF as they are able to further reduce the risk of stroke and systemic embolism compared to the previous standard of care, VKAs, and also lower the risk of intracranial hemorrhage. ^62^ However, given the hemostatic changes in patients with cirrhosis and the potential effect of liver dysfunction on drug pharmacokinetics, anticoagulation can be challenging in these patients. As a consequence, only a minority of patients with cirrhosis and NVAF—who have an indication for anticoagulation—receive anticoagulant drugs. However, in parallel with worldwide trends albeit to a lower extent, the proportion of patients with cirrhosis and NVAF receiving anticoagulants has also increased in recent years (from 39% in 2012 to 49% in 2019 in a US nationwide insurance claims database). ^63^

There are only a few studies—mainly with an observational retrospective design—that evaluated anticoagulation specifically in patients with cirrhosis and NVAF, and have sometimes yielded contrasting results. A meta-analysis based on these studies suggests that, compared with no treatment, the benefit of anticoagulation in terms of stroke risk reduction greatly outweighs the risk of bleeding in patients with cirrhosis and NVAF. ^64^ DOACs showed similar efficacy compared to VKAs, with a lower risk of major bleeding, gastrointestinal bleeding, and intracranial hemorrhage. ^65,66^ However, most of these data are derived from cohorts of patients with cirrhosis with less severe liver dysfunction (Child-Pugh class A or B).

The choice of the anticoagulant drug depends also on its pharmacological properties. VKAs are challenging in this population, due to spontaneously prolonged PT-INR in advanced cirrhosis and a potentially low time within the therapeutic range. However, cirrhosis can also affect the pharmacology of the DOACs, since liver impairment is associated with decreased cytochrome activity (resulting in reduced hepatic clearance) and reduced synthesis of albumin (resulting in increased free drug fractions). Degrees of hepatic clearance and plasma protein binding differ among DOACs. Indeed, all DOACs are contraindicated in Child-Pugh class C, while rivaroxaban—and according to the US Food and Drug Administration labeling also edoxaban—is not recommended for patients with Child-Pugh class B, due to their higher degree of hepatic metabolism.

A recent guidance of the International Society on Thrombosis and Haemostasis recommends anticoagulation for patients with AF and Child-Pugh class A or B cirrhosis when the CHA_2_DS_2_-VASc score is ≥ 2 in males or ≥ 3 in females, and suggests anticoagulation when the CHA_2_DS_2_-VASc score is 1 in males or 2 in females. ^67^ DOACs at standard doses are suggested over VKAs, due to their greater safety and efficacy profile. ^67^ No recommendation was available regarding Child-Pugh class C, since the evidence on the risk and benefit of anticoagulation in these patients is limited. However, this guidance was published before the ESC updated guidelines on NVAF suggesting the new CHA_2_DS_2_-VA score. ^60^

Apart from anticoagulation, alternative strategies can be considered in patients with more advanced liver cirrhosis. Although the latest ESC guidelines ^60^ recommend continuing oral anticoagulation indefinitely after AF ablation when indicated by the CHA_2_DS_2_-VA score, discontinuation after successful rhythm control with catheter ablation can be an option after two randomized controlled trials ^68,69^ reported very low rates of thromboembolic events in patients receiving either no therapy or aspirin. Percutaneous left atrial appendage occlusion can be considered in patients with contraindications to anticoagulant treatment, since it was associated with low rates of stroke with single antiplatelet therapy or even no antithrombotic treatment. ^60^

The therapeutic strategies for the treatment of deep vein thrombosis of the limbs and pulmonary embolism in patients with cirrhosis are similar to those suggested for patients with PVT and patients with AF. While LMWH and VKAs have long represented the cornerstone of treatment, an emerging role of DOACs as safe therapeutic alternatives has also emerged in this setting. ^47^ As opposed to patients with PVT, cirrhosis itself is usually not listed as a permanent risk factor for thrombosis when occurring in the lower limbs or in the pulmonary arteries, unless hepatocarcinoma is present. In case patients have an indication for extended secondary prevention, low-dose DOACs appear as an important therapeutic strategy to reduce the risk of bleeding complications. However, no studies have specifically addressed the role of low-dose DOACs in this setting. Like other patients with cirrhosis requiring anticoagulant treatment, we suggest a platelet count of 50× 10^9^/L as a possible threshold for maintaining standard-dose anticoagulation. Below this cut-off point, a case-by-case decision should be taken.

**Table:** 

KEYPOINTS for the management of AF • In patients with AF and Child-Pugh class A or B cirrhosis, anticoagulation is recommended when CHA_2_DS_2_-VA score ≥ 2 points and suggested when CHA_2_DS_2_-VA score = 1 point. • In patients with AF and Child-Pugh class C, due to limited data, a case-by-case evaluation is suggested. • DOACs are generally preferred over VKA: in Child-Pugh class A any DOAC can be used; in Child-Pugh class B DOACs should be used with caution (rivaroxaban and edoxaban are not recommended); in Child-Pugh class C, all DOACs are contraindicated. • Left atrial appendage occlusion can be considered when anticoagulation is contraindicated.	
